# Our Duty to Our Fellow Workers

**Published:** 1919-03-15

**Authors:** 


### HOSPITAL WEAKNESS & HOSPITAL STRENGTH

### OUR DUTY TO OUR FELLOW WORKERS.

Of late there has been much and continuous discussion of subjects of grave importance to the well-being and treatment of members of nursing staffs and nurses generally. The advent of the College of Nursing has given members of this profession new hope, larger ambitions and increased opportunities whereby they can, by discretion and courage, improve their position and enhance their emoluments. For years and years, owing in no small measure to the volume of work involved and to other causes, it has been the fashion for a matron to confine herself to the work she may be called upon to do in the particular hospital where she is employed. The College of Nursing has however, by steady propaganda and hard work, gradually induced the nurses individually to examine into the conditions under which they labour, are paid, fed and housed. These developments we have welcomed in The Hospital as vital to the uplifting, extension of influence and thorough training and instruction of those who are ambitious to become fully trained nurses. The introduction of the Sister-Tutor in the best schools in this country is effecting what practically amounts to a revolution in methods of training, and to a great uplifting of the status and educational facilities which were formerly open to pupil nurses. It has further led women of education to enter one of the few nurse-training schools which are pioneers of the new system and have able and educated women occupying the position of Sister-Tutor, some of them being in fact members of a University. The changes here indicated have been welcomed by the best of women throughout the country, and their action in joining the nursing schools has led the public and the medical profession to look forward with pleasurable anticipation to the time when it will be possible to obtain the services of a well educated lady, who is also a fully trained nurse, to occupy the position and carry on at their best the principles which Florence Nightingale laid down as essential to the highest and best success open to women to win.

The enemies of the College of Nursing, to the advent of which we owe many of the advances just referred to, have been in the habit of proclaiming themselves as the friends of nurses and then pointing with pride to the fact that for thirty years they have adovcated the State Registration of certificated nurses, though they have succeeded in accomplishing nothing practical to hasten the attainment of this end. The leaders of this party began by endeavouring to rule the nursing world by what amounted to a system of terrorism, by the use of big words and continuous and repeated denunciation of those to whom the nation owes in the largest measure the trained nurse as she exists in this country to-day. This system of abuse has been promulgated by a few women who have associated themselves together; as their utterances demonstrate, some possess an inadequate sense of responsibility, or none at all. Then, by repeatedly writing under this name or that to a particular publication, they have striven to make these utterances an instrument of denunciation and terror to the timid. This small group has associated together to provide copy for a print which is suspect as to the methods pursued, which would be manifestly impossible for lovers of truth with a sense of fairness and decorum. This print has been circulated for many years, twenty at least, and marked copies have been sent out to all sorts of people who, it was hoped, might be influenced by the violence of the language employed towards their selected victims who, when timid folk, have been frightened off opposition to the outrageous policy often pursued.

The real leaders of nursing opinion and the principal supporters of the College have heretofore treated this print and its fulminations with the contempt which they deserve. This courageous policy of opposition to the enemies of the College has put heart and courage into a continuously increasing number of trained nurses who are working women of considerable ability and alertness, old enough and experienced enough to know that to win a great battle in the face of such unworthy opponents must mean constant, self-sacrificing effort in defence of the highest principles. Such effort must, in the end, certainly secure the grateful recognition and support of all eminent, able and knowledgeable men and women throughout the country. Recently, growing disheartened by the small success which has attended their efforts, and the discrediting of their fulminations by the exposure which has befallen such contemptible tactics, and led to the demonstration of the hollowness and impotence of the authority and influence which these people have striven to make the world believe they possess, some enemies of the College have adopted a new course in desperation. This course is foreign to the accepted principles which govern the actions of all people in this country who hold their own reputation and the purity of their motives to be the real foundation upon which, by continuous hard work, the British public learns to know and trust public workers of the first rank. These services have often proved invaluable not only to the interests of individual workers, but to the State, the nation and the public generally. Necessity is said to know no law, and self-respect in handling public matters may be measured by the value attached by each individual lady or gentleman to truth, honour, and justice to his or her contemporaries. Instances have occurred where an honoured name, won by lifelong and zealous work for others, a just appreciation of this fact and of the services rendered, and even the recognition of the silence which we all owe to our dead contemporaries, have been

**Hospital Weakness and Hospital Strength**—(*cont.*)

ignored, and instead insult, insinuation and suggestion have been employed. These are so shameless and abominable as to make it essential that every working nurse throughout the land shall resolve in her heart to have nothing to do with such people who pose as leaders and defenders of British trained nurses, but may show by their utterances and conduct that they belong to a type and class of person which every community, in self-defence and honour, should oppose and expose. These types of opponents, whilst posing as leaders, do but foul the nurses' nest, especially when the methods pursued injure the good name of the trained nurses.

Some recent cases are notorious. Many nurses we know have been pained to the quick by, and properly regard, the conduct of the culprits in question as unworthy of womanhood. If they had sufficient importance, which they have not, their proceedings would be denounced as a disgrace to the British nation. We have thought it essential to call attention to this matter, and we shall be glad to receive communications from our readers of all types, doctors, matrons, sisters, nurses, members of hospital committees, supporters of hospitals, every one interested in our hospitals and the nursing profession, so that concerted steps may be taken, if and when opportunity may arise, to bring the offenders to book.

Further, the trained nurse everywhere should be placed in a position to pursue energetically her work for her profession, its development and improvement. The paramount duty of every trained nurse at this time is to join hands with her Sisters on the College Register to-day, and jointly pursue an active canvass to increase without fail and with as little delay as possible, the number of trained nurses on the College Register to 20,000 or 30,000 ardent supporters of the very best type. Then, in addition to the Press, in the right and proper meaning of the word, every trained nurse, when her name has been placed on the College Register, will have behind her an organisation and status which must bring her material advantages and improve her position immeasurably.

